# Postoperative central venous pressure is associated with acute kidney injury in patients undergoing coronary artery bypass grafting

**DOI:** 10.3389/fcvm.2022.1016436

**Published:** 2022-11-17

**Authors:** Jiale Li, Ruiling Wang, Jun Wan, Peng Zhu, Zezhou Xiao, Xiaowu Wang, Shaoyi Zheng

**Affiliations:** ^1^Department of Cardiovascular Surgery, Nanfang Hospital, Southern Medical University, Guangzhou, China; ^2^Department of Cardiovascular Surgery, Zhujiang Hospital, Southern Medical University, Guangzhou, China

**Keywords:** coronary artery bypass grafting, venous congestion, acute kidney injury, central venous pressure, mortality

## Abstract

**Objective:**

The present study aimed to investigate the association of postoperative central venous pressure (CVP) with acute kidney injury (AKI) and mortality in patients undergoing coronary artery bypass grafting (CABG).

**Method:**

Patients who underwent CABG in the MIMIC-III database were included and divided into two groups according to the optimal cutoff value of CVP for postoperative AKI determined by the receiver operating characteristic (ROC) curves. The association of CVP with AKI and mortality was determined by multivariate regression models. A 1:1 propensity score matching (PSM) was performed to balance the influence of potential confounding factors.

**Results:**

A total of 3,564 patients were included and divided into High CVP group (CVP ≥ 10.9 mmHg) and Low CVP group (CVP < 10.9 mmHg) according to the ROC analysis. Patients in High CVP group presented with higher AKI incidence (420 (28.2%) vs. 349 (16.8), *p* < 0.001), in-hospital mortality (28 (1.9%) vs. 6 (0.3%), *p* < 0.001) and 4-year mortality (149 (15.8%) vs. 162 (11.1%), *p* = 0.001). Multivariate regression model showed that CVP was an independent risk factor for the postoperative AKI (OR: 1.071 (1.035, 1.109), *p* < 0.001), in-hospital mortality (OR: 1.187 (1.026, 1.373), *p* = 0.021) and 4-year mortality (HR: 1.049 (1.003, 1.096), *p* = 0.035). A CVP above 10.9 mmHg was significantly associated with about 50% higher risk of AKI (OR: 1.499 (1.231, 1.824), *p* < 0.001). After PSM, 1004 pairs of score-matched patients were generated. The multivariate logistic model showed that patients with CVP ≥ 10.9 mmHg had a significantly higher risk of AKI (OR: 1.600 (1.268, 2.018), *p* < 0.001) in the PSM subset. However, CVP, as a continuous or a dichotomic variable, was not independently associated with in-hospital mortality (OR: 1.202 (0.882, 1.637), *p* = 0.244; OR: 2.636 (0.399, 17.410), *p* = 0.314) and 4-year mortality (HR: 1.030 (0.974, 1.090), *p* = 0.297; HR: 1.262 (0.911, 1.749), *p* = 0.162) in the PSM dataset.

**Conclusion:**

A mean CVP ≥ 10.9 mmHg within the first 24 h after CABG was independently associated with a higher risk of postoperative AKI.

## Introduction

Acute kidney injury (AKI) is a common postoperative complication in patients undergoing coronary artery bypass grafting (CABG), of which the incidence is about 37%. It is independently associated with both short-term and long-term mortality (1). Previously, hypoperfusion has been considered as the main hemodynamic determinant of AKI. Both hypovolemia and hypotension, especially complicated with cardiac dysfunction, can lead to the insufficient blood supply for the kidneys (2–4). In consequence, clinicians pay more attention to the blood pressure and cardiac output for adequate renal perfusion. However, recent studies have raised the concept that venous congestion also makes great contribution to the development of AKI (1–5). Venous congestion can lead to systemic venous hypertension and increase the central venous pressure (CVP). Due to the low venous resistance, the renal venous pressure can be raised with the increased CVP (6). Venous congestion can reduce kidney arteriovenous pressure gradient, increase intrarenal pressure, activate sympathetic system and renin-angiotensin-aldosterone system, and consequently lead to the reduction of renal blood flow, lower glomerular filtration rate, and retention of water and sodium. CVP is routinely used to evaluate the body volume status and cardiac function in the early postoperative period of CABG. Whereas, the prognostic value of postoperative CVP on the incidence of AKI and mortality in CABG patients has not been intensively studied. Therefore, the objective of the present study was to investigate the association between postoperative CVP and AKI and mortality in patients undergoing CABG.

## Materials and methods

### Patient selection

A retrospective cohort study was conducted based on the Medical Information Mart for Intensive Care III (MIMIC-III) database (v1.4). The MIMIC-III database is a large, freely available database with more than 40,000 patients who had intensive care unit (ICU) stays at the Beth Israel Deaconess Medical Center between 2001 and 2012. In the MIMIC-III database, clinical data were collected from two different clinical information systems: CareVue and MetaVision. The MIMIC-III database contains dates of death up to 90 days in the future for Metavision patients and up to 4 years in the future for CareVue patients (7). Authors (JL, RW) had obtained access to the database and was responsible for data extraction. Our study complied with the Reporting of Studies Conducted using Observational Routinely Collected Health Data statement.

Patients in the MIMIC-III database undergoing CABG and admitted to the cardiac surgery recovery unit (CSRU) were eligible for inclusion. CABG procedure was identified based on the ICD9-CM (International Classification of Diseases, Ninth Revision, Clinical Modification) code in the MIMIC-III database (3610-3616). For those with multiple hospital admission for CABG in the MIMIC-III database, only the first admission was analyzed. We excluded patients who were younger than 18 years old and those complicated with end-stage kidney disease (ICD9-CM code: 5856) or stage 5 chronic kidney disease (ICD9-CM code: 5855). We excluded patients without CVP measurement within the first 24 h after CSRU admission, as well as patients without creatinine measurement on admission or after CSRU admission. Patients who underwent concomitant valvular (ICD9-CM code: 3520-3528, 3599, 3510-3514) and/or aortic surgery (ICD9-CM code: 3804, 3814, 3834, 3844, 3864, 3884, 3971, 3973, 3978) were also excluded.

### Variable extraction

Clinical characteristics were collected using structured query language, including demographics (age, gender, body mass index (BMI), comorbidities (cardiac arrhythmias, congestive heart failure, peripheral vascular disease, myocardial infarction, chronic pulmonary disease, liver disease, renal disease, diabetes mellitus), laboratory events on admission (blood urea nitrogen (BUN), creatinine, white blood cell, platelet, hemoglobin), surgery-related variables (cardiopulmonary bypass (CPB), number of revascularized coronary artery and number of internal mammary artery (IMA) used for bypass grafting), as well as vital signs (CVP, heart rate, systolic blood pressure (SBP), diastolic blood pressure (DBP), mean blood pressure (MBP), respiratory rate, oxygen saturation (SPO_2_), erythrocyte transfusion, net fluid input (input minus output) and SOFA (Sequential organ failure assessment) score within the first 24 h after CSRU admission. Outcomes including ventilation duration, length of stay in CSRU, postoperative AKI, and mortality were also collected. In the present study, the time of CSRU admission was considered as that of the end of the operation. The mean value of laboratory variables on admission and vitals (∼1 data point per hour) within the first 24 h after CSRU admission were calculated and used in the present study. Comorbidities were collected based on the ICD9-CM code in the MIMIC-III database (8) and the Charlson comorbidity index (CCI) was also calculated. Creatinine was used to determine the incidence of AKI according to the Kidney Disease Improving Global Outcomes (KDIGO) criteria (9).

### Outcome

The primary outcome in the present study was the incidence of postoperative AKI and the secondary outcome included in-hospital and 4-year mortality.

### Statistical analysis

Data were presented as means (standard deviations) or medians (interquartile ranges) for continuous variables, and total numbers (percentages) for categorical variables. Receiver operating characteristic (ROC) curves were constructed and used to determine the optimal cutoff value of CVP for the incidence of AKI according to the Youden index. Thereby, patients were divided into two groups. As appropriate, comparisons between groups were made through the Student-t test or Mann-Whitney U test for continuous variables, and the Chi-square test or Fisher’s exact test for categorical variables. Survival curves were estimated using the Kaplan-Meier method and compared by the log-rank test. Logistic regression was used to characterize the relationship of CVP with AKI and in-hospital mortality, while Cox regression was selected to determine the association of CVP with 4-year mortality. The adjusted covariates were selected based on previously published studies and clinically relevant experience. Different models were constructed to investigate the effects of different confounders on the association between CVP and outcomes (Enter). Model 1 was adjusted for demographics including age, gender, and BMI; Model 2 was adjusted for age, gender, BMI, comorbidities, CCI and laboratory events on admission; Model 3 was adjusted for the variables in Model 2 plus surgery-related variables; Model 4 was adjusted for the variables in Model 3 plus variables related to the clinical situation of patients within the first 24 h after CSRU admission, including vitals, erythrocyte transfusion, net fluid input and SOFA score. The results were presented as odds ratios (ORs) or hazard ratios (HRs), and 95% confidence intervals (CIs). Variables of the number of revascularized coronary artery and the number of IMA used for bypass grafting were selected as continuous variables. The analyses for 4-year mortality were restricted to patients in the CareVue system.

Propensity score matching (PSM) was used to balance the potential confounding factors to ensure the robustness of our findings. Based on the multivariate logistic regression model, the propensity score for CVP value was calculated according to the baseline characteristics, including demographics, comorbidities, CCI, laboratory events on admission, surgery-related variables, as well as vital signs, erythrocyte transfusion, net fluid input and SOFA score within the first 24 h after CSRU admission. Then patients were matched based on the propensity score with a caliper width of 0.02.

All tests were two-sided, and *p* < 0.05 were considered significant. All statistical analyses in our study were performed using SPSS Statistics 23 (IBM, Chicago, IL).

## Results

### Baseline characteristics

**FIGURE 1:**
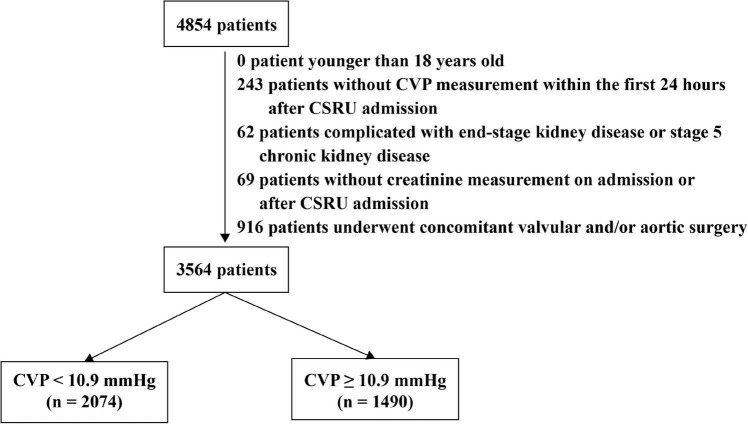
The flow diagram of patient selection. Patients were divided into two groups according to the results of receiver operating characteristic curves. CSRU: Cardiac surgery recovery unit. CVP: Central venous pressure.

The flow diagram of patient selection is presented in Figure 1. A total of 4,854 patients undergoing CABG and admitted to the CSRU after surgery were identified in the MIMIC-III database, of whom 3,564 were included in the present study. The clinical characteristics of those patients were presented in Table 1. The median of CVP within the first 24 h after CSRU admission was 10.26 (8.27, 12.48) mmHg. The AKI incidence was 21.6% and the in-hospital mortality was 1.0%. The 4-year mortality was 13.0% among 2,395 patients in the CareVue system.

**TABLE 1:** Clinical characteristics of patients undergoing CABG.

	Before PSM (*n* = 3,564)	After PSM (*n* = 2,008)
**Demographics**		
Age	67.10 (59.39, 74.37)	66.25 (58.49, 73.70)
Gender		
Male	2,736 (76.8)	1554 (77.4)
Female	828 (23.2)	454 (22.6)
BMI	28.22 (25.13, 31.94)	28.93 (25.85, 32.29)
**Comorbidities**		
Cardiac arrhythmia	1,510 (42.4)	829 (41.3)
Myocardial infarction	1,342 (37.7)	766 (38.1)
Congestive heart failure	692 (19.4)	379 (18.9)
Peripheral vascular disease	452 (12.7)	249 (12.4)
Chronic pulmonary disease	540 (15.2)	298 (14.8)
Liver disease	82 (2.3)	42 (2.1)
Diabetes mellitus	1,439 (40.4)	855 (42.6)
Kidney disease	302 (8.5)	171 (8.5)
**CCI**	1.00 (1.00, 2.00)	1.00 (1.00, 2.00)
Laboratory examination*		
Hemoglobin (g/dL)	11.37 (10.13, 12.85)	11.38 (10.13, 12.95)
White blood cell (× 10^9^/L)	9.50 (7.40, 12.40)	9.50 (7.40, 12.70)
Platelet (× 10^9^/L)	196.00 (158.45, 244.00)	196.50 (158.00, 243.50)
Creatinine (mg/dL)	0.90 (0.78, 1.10)	0.93 (0.80, 1.10)
BUN (mg/dL)	16.67 (13.50, 21.50)	16.67 (13.50, 21.50)
**Surgery**		
CPB	3315 (93)	1865 (92.9)
Revascularized arteries^&^	2.00 (2.00, 3.00)	2.00 (2.00, 3.00)
1	545 (15.3)	307 (15.3)
2	1451 (40.7)	793 (39.5)
3	1231 (34.5)	701 (34.9)
≥ 4	337 (9.5)	207 (10.3)
IMA^&&^	1.00 (1.00, 1.00)	1.00 (1.00, 1.00)
0	216 (6.1)	118 (5.9)
1	3303 (92.7)	1863 (92.8)
2	45 (1.3)	27 (1.3)
Vital signs**		
CVP (mmHg)	10.26 (8.27, 12.48)	10.89 (8.95, 12.63)
CVP <10.9 mmHg	2074 (58.2)	1004 (50)
CVP ≥ 10.9 mmHg	1490 (41.8)	1004 (50)
Heart rate (bpm)	85.58 (80.11, 91.56)	85.92 (80.63, 91.82)
SBP (mmHg)	111.40 (106.36, 117.90)	110.99 (106.11, 116.85)
DBP (mmHg)	56.36 (52.65, 60.36)	56.75 (52.97, 60.76)
MBP (mmHg)	74.26 (70.88, 78.05)	74.34 (70.91, 78.21)
Respiratory rate (bpm)	16.67 (15.13, 18.50)	16.83 (15.23, 18.75)
SpO_2_ (%)	98.29 (97.42, 99.03)	98.18 (97.29, 98.97)
Erythrocyte transfusion**	0 (0, 350.00)	0 (0, 350.00)
Net fluid input**	978.00 (-72.50, 2132.00)	1033 (-49.00, 2174.75)
SOFA score**	3.00 (2.00, 5.00)	3.00 (2.00, 5.00)
**Outcome**		
Ventilation duration	5.00 (2.87, 12.64)	5.03 (2.93, 12.18)
Length of stay in CSRU	2.05 (1.19, 3.21)	2.06 (1.21, 3.15)
AKI	769 (21.6)	410 (20.4)
AKI staging		
1	633 (17.8)	346 (17.2)
2	81 (2.3)	40 (2.0)
3	55 (1.5)	24 (1.2)
In-hospital mortality	34 (1.0)	12 (0.6)
4-year mortality^†^	311 (13.0)	158 (12.2)

Data were presented as means (standard deviations) or medians (interquartile ranges) for continuous variables, and total numbers (percentages) for categorical variables. AKI: Acute kidney injury. BMI: Body mass index. BUN: Blood urea nitrogen. CABG: Coronary artery bypass grafting. CCI: Charlson comorbidity index. CPB: Cardiopulmonary bypass. CSRU: Cardiac surgery recovery unit. CVP: Central venous pressure. DBP: Diastolic blood pressure. IMA: Internal mammary artery. MBP: Mean blood pressure. PSM: Propensity score matching. SBP: Systolic blood pressure. SOFA: Sequential organ failure assessment.

*Variables on admission.

**Variables within the first 24 h after CSRU admission.

^&^Number of revascularized coronary arteries.

^&&^Number of IMA used for bypass grafting.

^†^Limited to patients in the CareVue system.

**FIGURE 2:**
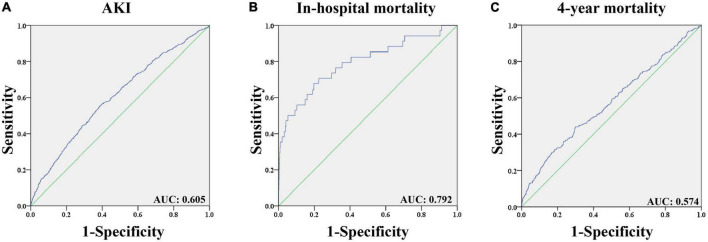
The receiver operating characteristic curves of predictive value of CVP for outcomes in patients undergoing CABG. **(A)** Predictive value of CVP for AKI; **(B)** Predictive value of CVP for in-hospital mortality; **(C)** Predictive value of CVP for 4-year mortality. AKI: Acute kidney injury. AUC: Area under the curve. CABG: Coronary artery bypass grafting. CVP: Central venous pressure.

ROC analysis (Figure 2) was performed to assess the predictive value of CVP for postoperative AKI, and the area under the curve (AUC) was 0.605 (*p* < 0.001). The optimal cutoff value of CVP for postoperative AKI was 10.9 mmHg with a sensitivity of 0.553 and a specificity of 0.614. Additionally, it was found that CVP had a strong discriminatory ability for the in-hospital mortality (AUC: 0.792, *p* < 0.001), but unsatisfactory performance regarding the 4-year mortality (AUC: 0.574, *p* < 0.001).

Based on the results of ROC analysis, patients were divided into two groups according to the CVP value: High CVP group (CVP ≥ 10.9 mmHg; n = 1490) and Low CVP group (CVP < 10.9 mmHg; n = 2074). Besides, 942 (63.2%) patients in the High CVP group and 1,453 (70.1%) patients in the Low CVP group were from CareVue system. As shown in Table 2, significant differences in baseline characteristics could be observed between the two groups. Patients in the High CVP group were more likely to be younger and female. They had significantly higher BMI, BUN, white blood cell count, platelet, and creatinine on admission. Also, they tended to have more comorbidities. Within the first 24 h after CSRU admission, patients with higher CVP presented with higher heart rates and diastolic blood pressure, as well as lower systolic blood pressure and oxygen saturation. They also tended to receive higher volume of erythrocyte transfusion and net fluid input and have higher SOFA score after the first 24 h in CSRU.

**TABLE 2:** Clinical characteristics of patients in different groups.

	Before PSM			After PSM		
	Low CVP group (*n* = 2074)	High CVP group (*n* = 1490)	*p*	Low CVP group (*n* = 1004)	High CVP group (*n* = 1004)	*p*
**Demographics**						
Age	67.80 (60.43, 74.99)	66.16 (58.31, 73.67)	<0.001	66.04 (58.49, 73.44)	66.49 (58.47, 74.03)	0.572
Gender			0.016			0.831
Male	1622 (78.2)	1114 (74.8)		775 (77.2)	779 (77.6)	
Female	452 (21.8)	376 (25.2)		229 (22.8)	225 (22.4)	
BMI	27.14 (24.40, 30.45)	29.90 (26.72, 33.98)	<0.001	28.81 (25.70, 32.28)	29.03 (26.17, 32.31)	0.393
**Comorbidities**						
Cardiac arrhythmia	837 (40.4)	673 (45.2)	0.004	412 (41.0)	417 (41.5)	0.821
Myocardial infarction	696 (33.6)	646 (43.4)	<0.001	377(37.5)	389 (38.7)	0.581
Congestive heart failure	318 (15.3)	374 (25.1)	<0.001	184(18.3)	195 (19.4)	0.530
Peripheral vascular disease	257 (12.4)	195 (13.1)	0.538	125 (12.5)	124 (12.4)	0.946
Chronic pulmonary disease	272 (13.1)	268 (18.0)	<0.001	147(14.6)	151 (15.0)	0.802
Liver disease	34 (1.6)	48 (3.2)	0.002	20 (2.0)	22 (2.2)	0.755
Diabetes mellitus	770 (37.1)	669 (44.9)	<0.001	430(42.8)	425 (42.3)	0.821
Kidney disease	143 (6.9)	159 (10.7)	<0.001	85 (8.5)	86 (8.6)	0.936
**CCI**	1.00 (0, 2.00)	2.00 (1.00, 3.00)	<0.001	1.00 (1.00, 2.00)	1.00 (1.00, 2.00)	0.342
Laboratory examination*						
Hemoglobin (g/dL)	11.40 (10.17, 12.90)	11.33 (10.10, 12.80)	0.489	11.45 (10.20, 13.00)	11.29 (10.10, 12.89)	0.377
White blood cell (×10^9^/L)	9.40 (7.27, 12.28)	9.60 (7.55, 12.65)	0.007	9.40 (7.30, 12.75)	9.60 (7.50, 12.66)	0.184
Platelet (×10^9^/L)	193.33 (157.50, 238.00)	200.33 (160.27, 253.00)	0.001	195.50 (158.54, 241.00)	197.00 (157.00, 249.00)	0.441
Creatinine (mg/dL)	0.9 (0.75, 1.10)	0.95 (0.80, 1.17)	<0.001	0.90 (0.80, 1.10)	0.95 (0.80, 1.10)	0.162
BUN (mg/dL)	16.50 (13.00, 20.75)	17.00 (13.67, 22.67)	<0.001	17.00 (13.50, 21.50)	16.67 (13.50, 21.50)	0.754
**Surgery**						
CPB	1,933 (93.2)	1382 (92.8)	0.603	937 (93.3)	928 (92.4)	0.435
Revascularized arteries^&^	2.00 (2.00, 3.00)	2.00 (2.00, 3.00)	0.101	2.00 (2.00, 3.00)	2.00 (2.00, 3.00)	0.234
1	327 (15.8)	218 (14.6)		148 (14.7)	159 (15.8)	
2	855 (41.2)	596 (40.0)		385 (38.3)	408 (40.6)	
3	708 (34.1)	523 (35.1)		370 (36.9)	331 (33.0)	
≥ 4	184 (8.9)	153 (10.3)		101 (10.1)	106 (10.6)	
IMA^&&^	1.00 (1.00, 1.00)	1.00 (1.00, 1.00)	0.016	1.00 (1.00, 1.00)	1.00 (1.00, 1.00)	0.948
0	104 (5.0)	112 (7.5)		57 (5.7)	61 (6.1)	
1	1947 (93.9)	1356 (91.0)		936 (93.2)	927 (92.3)	
2	23 (1.1)	22 (1.5)		11 (1.1)	16 (1.6)	
Vital signs**						
CVP (mmHg)	8.63 (7.27, 9.77)	12.88 (11.84, 14.35)	<0.001	8.96 (7.65, 9.96)	12.63 (11.67, 13.85)	<0.001
Heart rate (bpm)	84.96 (79.66, 90.63)	86.43 (80.73, 93.33)	<0.001	86.00 (80.72, 91.33)	85.87 (80.37, 92.38)	0.663
SBP (mmHg)	112.27 (106.96, 119.08)	110.28 (105.60, 115.94)	<0.001	111.01 (106.04, 117.16)	110.96 (106.24, 116.58)	0.995
DBP (mmHg)	55.91 (52.29, 59.79)	56.92 (53.18, 61.11)	<0.001	56.70 (52.85, 60.50)	53.13 (56.78, 60.83)	0.427
MBP (mmHg)	74.14 (70.82, 77.83)	74.49 (70.94, 78.29)	0.067	74.24 (70.83, 78.16)	74.49 (70.95, 78.29)	0.341
Respiratory rate (bpm)	16.58 (15.00, 18.42)	16.79 (15.29, 18.63)	0.004	16.92 (15.22, 18.83)	16.76 (15.26, 18.60)	0.731
SpO_2_ (%)	98.39 (97.51, 99.11)	98.17 (97.31, 98.94)	<0.001	98.18 (97.23, 98.97)	98.18 (97.38, 98.97)	0.829
Erythrocyte transfusion**	0 (0, 0)	0 (0, 375)	<0.001	0 (0, 350)	0 (0, 350)	0.441
Net fluid input**	762.00 (−198.00, 1811.00)	1302.00 (142.00, 2591.00)	<0.001	1019.50 (−36.50, 2085.00)	1059.00 (−72.00, 2198.00)	0.612
SOFA score**	3.00 (2.00, 4.00)	4.00 (3.00, 6.00)	<0.001	3.00 (2.00, 5.00)	3.00 (2.00, 5.00)	0.807
**Outcome**						
Ventilation duration	4.00 (2.50, 7.90)	7.73 (3.67, 18.44)	<0.001	4.33 (2.57, 9.13)	6.00 (3.25, 14.27)	<0.001
Length of stay in CSRU	1.66 (1.14, 2.84)	2.29 (1.29, 4.12)	<0.001	1.97 (1.18, 3.06)	2.14 (1.25, 3.28)	<0.001
AKI	349 (16.8)	420 (28.2)	<0.001	168(16.7)	242 (24.1)	<0.001
AKI staging						
1	306 (14.8)	327 (21.9)		141 (14.0)	205 (20.4)	
2	24 (1.2)	57 (3.8)		15 (1.5)	25 (2.5)	
3	19 (0.9)	36 (2.4)		12 (1.2)	12 (1.2)	
In-hospital mortality	6 (0.3)	28 (1.9)	<0.001	3 (0.3)	9 (0.9)	0.082
4-year mortality^†^	162 (11.1)	149 (15.8)	0.001	73 (10.9)	85 (13.5)	0.161

Data were presented as means (standard deviations) or medians (interquartile ranges) for continuous variables, and total numbers (percentages) for categorical variables. AKI, Acute kidney injury; BMI, Body mass index; BUN, Blood urea nitrogen; CABG, Coronary artery bypass grafting; CCI, Charlson comorbidity index; CPB, Cardiopulmonary bypass; CSRU, Cardiac surgery recovery unit; CVP, Central venous pressure; DBP, Diastolic blood pressure; IMA, Internal mammary artery; MBP, Mean blood pressure; PSM, Propensity score matching; SBP, Systolic blood pressure; SOFA, Sequential organ failure assessment.

*Variables on admission.

**Variables within the first 24 h after CSRU admission.

^&^Number of revascularized coronary arteries.

^&&^Number of IMA used for bypass grafting.

^†^Limited to patients in the CareVue system.

### Outcome

**FIGURE 3:**
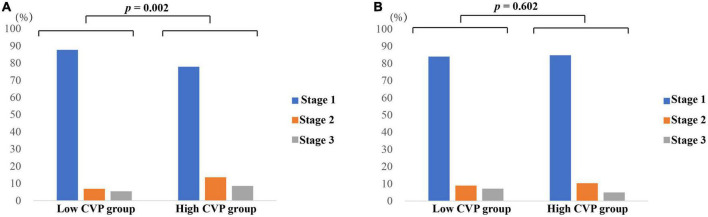
AKI staging of patients in different groups. **(A)** AKI staging of patients before PSM; **(B)** AKI staging of patients after PSM. *p* value was calculated by Chi square test and indicated in the plots. AKI, Acute kidney injury. CABG, Coronary artery bypass grafting. CVP, Central venous pressure.

**FIGURE 4:**
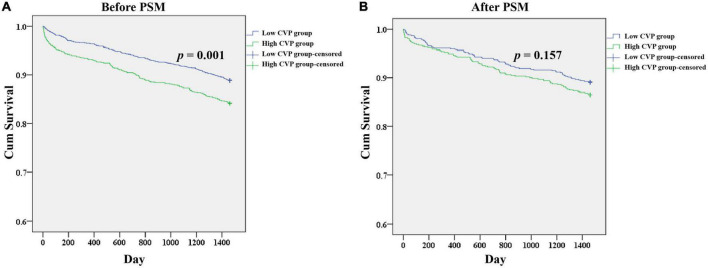
Kaplan-Meier survival analysis plots for 4-year overall survival in patients undergoing CABG. The analysis was limited to patients in the CareVue system. A significantly lower 4-year survival rate can be observed in the High CVP group in patients before PSM **(A)**, but not in patients after PSM **(B)**. *p* value was calculated by log-rank test and indicated in the plots. CABG, Coronary artery bypass grafting; CVP, Central venous pressure; PSM, propensity score matching.

As shown in Table 2, patients in the High CVP group had significantly longer ventilation duration (7.73 (3.67, 18.44) vs. 4.00 (2.50, 7.90), *p* < 0.001) and length of stay in CSRU (2.29 (1.29, 4.12) vs. 1.66 (1.14, 2.84), *p* < 0.001). The AKI incidence was significantly higher in the High CVP group (420 (28.2%) vs. 349 (16.8), *p* < 0.001) and the AKI was likely to be more severe (*p* = 0.002; Figure 3). Patients in the High CVP group had a significantly higher in-hospital mortality (28 (1.9%) vs. 6 (0.3%), *p* < 0.001) and 4-year mortality (149 (15.8%) vs. 162 (11.1%), *p* = 0.001) compared to those in the Low CVP group (Table 2). The Kaplan-Meier survival curves comparing the 4-year mortality of the two groups are shown in Figure 4. High CVP group showed significantly lower 4-year survival (*p* = 0.001).

**FIGURE 5:**
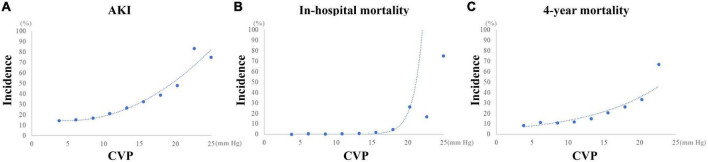
Curvilinear relationship between CVP and the outcomes of CABG patients. **(A)** Incidence of AKI. **(B)** Incidence of in-hospital mortality. **(C)** Incidence of 4-year mortality. AKI, Acute kidney injury; CABG, Coronary artery bypass grafting; CVP, Central venous pressure.

The curvilinear relationship between CVP and incidence of AKI is presented in Figure 5. The incidence of AKI increased as CVP increased, especially when CVP was higher than approximately 10 mmHg. After full adjustment, the logistic regression model showed that CVP was an independent risk factor for the incidence of postoperative AKI (OR: 1.071 (1.035, 1.109), *p* < 0.001; Table 3). Especially, a CVP above 10.9 mmHg was significantly associated with about 50% higher risk of AKI (OR: 1.499 (1.231, 1.824), *p* < 0.001).

**TABLE 3:** Association of postoperative CVP with AKI and mortality.

Outcome	Variable	OR/HR (95%CI)				
		Unadjusted	Model 1	Model 2	Model 3	Model 4
AKI	CVP (mmHg)	1.132 (1.103, 1.161)	1.128 (1.098, 1.160)	1.110 (1.078, 1.143)	1.108 (1.076, 1.141)	1.071 (1.035, 1.109)
	CVP ≥ 10.9 mmHg	1.940 (1.651, 2.279)	1.951 (1.643, 2.316)	1.800 (1.502, 2.157)	1.785 (1.489, 2.139)	1.499 (1.231, 1.824)
In-hospital mortality	CVP (mmHg)	1.467 (1.345, 1.601)	1.445 (1.329, 1.594)	1.356 (1.217, 1.513)	1.332 (1.196, 1.483)	1.187 (1.026, 1.373)
	CVP ≥ 10.9 mmHg	6.601 (2.726, 15.982)	6.361 (2.573, 15.726)	4.076 (1.560, 10.649)	3.989 (1.514, 10.514)	1.694 (0.543, 5.284)
4-year mortality	CVP (mmHg)	1.111 (1.072, 1.150)	1.130 (1.090, 1.172)	1.097 (1.057, 1.138)	1.091 (1.052, 1.132)	1.049 (1.003, 1.096)
	CVP ≥ 10.9 mmHg	1.473 (1.179, 1.840)	1.641 (1.302, 2.069)	1.489 (1.171, 1.893)	1.488 (1.170, 1.894)	1.160 (0.885, 1.521)

Model 1 adjusted for age, gender, BMI.

Model 2 adjusted for variables in Model 1, plus comorbidities (cardiac arrhythmias, congestive heart failure, peripheral vascular disease, myocardial infarction, chronic pulmonary disease, liver disease, renal disease, diabetes mellitus) and laboratory examinations (BUN, creatinine, white blood cell, platelet, hemoglobin) on admission.

Model 3 adjusted for variables in Model 2, plus surgery-related variables.

Model 4 adjusted for variables in Model 3, plus variables related to the clinical situation in CSRU, including vitals, SOFA score, net fluid input and erythrocyte transfusion within the first 24 h after CSRU admission.

AKI, Acute kidney injury; BMI, Body mass index; BUN, Blood urea nitrogen; CI, Confidence interval; CSRU, Cardiac surgery recovery unit; CVP, Central venous pressure; HR, Hazard ratio; OR, Odds ratio; SOFA, Sequential organ failure assessment.

As shown in Figure 5, after a plateau, the incidence of in-hospital mortality increased dramatically as CVP reached 16 mmHg approximately. Similarly, a mild increase of the incidence of 4-year mortality could be observed as CVP above 16 mmHg. As a continuous variable, the mean CVP within the first 24 h after surgery was independently related with the in-hospital mortality (OR: 1.187 (1.026, 1.373), *p* = 0.021) and 4-year mortality (HR: 1.049 (1.003, 1.096), *p* = 0.035) (Table 3). However, a CVP above 10.9 mmHg was not significantly associated with higher risk of in-hospital mortality (OR: 1.694 (0.543, 5.284), *p* = 0.364) and 4-year mortality (HR: 1.160 (0.885, 1.521), *p* = 0.283).

Similar results of multivariate regression analyses on subgroups of patients undergoing on-pump or off-pump CABG could be observed, as shown in the supplementary Tables 1, 2.

### The prognostic significance of postoperative central venous pressure after propensity score matching

Considering the imbalanced baseline characteristics between groups, we performed a 1:1 ratio PSM to balance the potential confounding factors, and 1,004 pairs of score-matched patients were generated. The baseline characteristics of patients after PSM are shown in Table 1. The AKI incidence was 20.4% and in-hospital mortality was 0.6%. The 4-year mortality was 12.2% among 1,199 patients in the CareVue system (631 (62.8%) in the High CVP group and 668 (66.5%) in the Low CVP group).

As shown in Table 2, the demographics, comorbidities, laboratory events on admission, surgery-related variables, as well as vital signs, erythrocyte transfusion, net fluid input and SOFA score within the first 24 h after CSRU admission were well balanced between the two groups. After PSM, significant differences between the two groups can still be observed in ventilation duration (6.00 (3.25, 14.27) vs. 4.33 (2.57, 9.13), *p* < 0.001), length of stay in CSRU (2.14 (1.25, 3.28) vs. 1.97 (1.18, 3.06), *p* < 0.001), and AKI incidence (242 (24.1%) vs. 168 (16.7%), *p* < 0.001). However, the differences of in-hospital mortality (9 (0.9%) vs. 3(0.3%), *p* = 0.082) and 4-year mortality (85 (13.5%) vs. 73 (10.9%), *p* = 0.161) between the two groups were not statistically significant. After PSM, patients in the High CVP group did not present with a tendency to be exposed to more severe AKI (*p* = 0.602; Figure 3). The Kaplan-Meier survival curves comparing patients in the two groups indicated that patients in the High CVP group had higher 4-year mortality, but the difference between the two groups did not reach statistical significance (*p* = 0.157) (Figure 4). After full adjustment, multivariate regression models indicated that after PSM CVP remained independently related with the incidence of postoperative AKI (OR: 1.090 (1.045, 1.138), *p* < 0.001; Table 4). Also, CVP above 10.9 mmHg was significantly associated with about 60% higher risk of AKI (OR: 1.600 (1.268, 2.018), *p* < 0.001). However, CVP, as a continuous or a dichotomic variable, was not independently associated with both in-hospital mortality (OR: 1.202 (0.882, 1.637), *p* = 0.244; OR: 2.636 (0.399, 17.410), *p* = 0.314) and 4-year mortality (HR: 1.030 (0.974, 1.090), *p* = 0.297; HR: 1.262 (0.911, 1.749), *p* = 0.162) in the PSM dataset (Table 4).

**TABLE 4:** Association of postoperative CVP with AKI and mortality after propensity score matching.

Outcome	Variable	OR/HR (95%CI)				
		Unadjusted	Model 1	Model 2	Model 3	Model 4
AKI	CVP (mmHg)	1.106 (1.063, 1.151)	1.100 (1.057, 1.145)	1.096 (1.052, 1.142)	1.096 (1.051, 1.142)	1.090 (1.045, 1.138)
	CVP ≥ 10.9 mmHg	1.580 (1.268, 1.969)	1.574 (1.260, 1.967)	1.584 (1.260, 1.990)	1.583 (1.260, 1.990)	1.600 (1.268, 2.018)
In-hospital mortality	CVP (mmHg)	1.474 (1.249, 1.740)	1.395 (1.179, 1.650)	1.355 (1.112, 1.650)	1.365 (1.123, 1.659)	1.202 (0.882, 1.637)
	CVP ≥ 10.9 mmHg	3.018 (0.815, 11.181)	2.867 (0.769, 10.686)	2.878 (0.728, 11.374)	3.110 (0.755, 12.804)	2.636 (0.399, 17.410)
4-year mortality	CVP (mmHg)	1.073 (1.013, 1.136)	1.068 (1.011, 1.128)	1.049 (0.993, 1.107)	1.048 (0.992, 1.107)	1.030 (0.974, 1.090)
	CVP ≥ 10.9 mmHg	1.253 (0.917, 1.713)	1.226 (0.896, 1.678)	1.273 (0.926, 1.750)	1.307 (0.946, 1.806)	1.262 (0.911, 1.749)

Model 1 adjusted for age, gender, BMI.

Model 2 adjusted for variables in Model 1, plus comorbidities (cardiac arrhythmias, congestive heart failure, peripheral vascular disease, myocardial infarction, chronic pulmonary disease, liver disease, renal disease, diabetes mellitus) and laboratory examinations (BUN, creatinine, white blood cell, platelet, hemoglobin) on admission.

Model 3 adjusted for variables in Model 2, plus surgery-related variables.

Model 4 adjusted for variables in Model 3, plus variables related to the clinical situation in CSRU, including vitals, SOFA score, net fluid input and erythrocyte transfusion within the first 24 h after CSRU admission.

AKI, Acute kidney injury; BMI, Body mass index; BUN, Blood urea nitrogen; CI, Confidence interval; CSRU, Cardiac surgery recovery unit; CVP, Central venous pressure; HR, Hazard ratio; OR, Odds ratio; SOFA, Sequential organ failure assessment.

## Discussion

Decreased renal perfusion has been considered as the main hemodynamic determinant of AKI after cardiac surgery (4, 10). Kidney is susceptible to ischemia due to the low density of vasculature in the renal medulla (11). To ensure sufficient renal perfusion for CABG patients, it is considered that adequate blood volume, blood pressure and cardiac output are necessary. Especially, MBP above 65 mmHg is a common target in the clinical practice to avoid AKI (12). However, there is not enough strong evidence to support that high level of MBP is an effective strategy to prevent the cardiac surgery-associated AKI (11, 13, 14). In line with the previous studies, MBP was not independently associated with AKI after CABG in the present study (data not shown).

Ongoing evidence showed that congestive renal failure precipitated by venous congestion is also an important factor contributing to the development of AKI in cardiac patients (1). Because of the low venous resistance, the increased CVP caused by venous congestion can be reversibly transmitted to the renal vein (1, 5, 6), leading to lower renal arteriovenous pressure gradient and higher intrarenal pressure (5, 15–18). Renal self-regulation is partially based on the renal arteriovenous pressure gradient. Therefore, the reduction of renal arteriovenous pressure gradient can compromise the renal self-regulation and consequently reduce the renal blood flow and glomerular filtration rate (5, 15–18). Also, the additive intrarenal pressure can result in a compression of renal vasculature and tubule, aggravating the renal hypoperfusion and urinary retention in the tubule (6). Except for the urinary retention in the renal tubule, the sympathetic nervous system and renin-angiotensin-aldosterone system, activated by the increased CVP and renal venous pressure, can promote the renal reabsorption of water and sodium and exacerbate the venous congestion (19, 20). Also, the activation of neuroendocrine system can further reduce the renal blood flow (21). Ganda et al. suggested that renal blood flow was reduced more by an increase of venous pressure than by such an equivalent decrease in arterial pressure (22). Damman et al. also reported that elevated venous pressure was an important determinant of renal function for patients with low renal blood flow (5). In 2008, a retrospective study showed that CVP was negatively associated with the glomerular filtration rate of patients with cardiovascular diseases (23). Stevenson et al. have introduced that in patients with chronic heart failure, the presence of venous congestion significantly decreased their 1-year survival rate. Complicated with low cardiac output, venous congestion could further worsen their prognosis (24). As aforementioned, adequate blood volume, blood pressure and cardiac output are necessary for sufficient renal perfusion after CABG (12). However, venous congestion is mainly the result of fluid overload and cardiac dysfunction, which are common postoperative complications of CABG patients and able to increase the level of CVP. It is likely that venous congestion is an important reason for the development of AKI after CABG and an elevated CVP level may indicate a higher risk of AKI.

Prior studies have identified CVP as an independent predictor of prognosis in patients with cardiovascular disease (23), patients with advanced decompensated heart failure (25), and patients undergoing lung transplantation (26), but evidence of the predictive value of postoperative CVP in patients undergoing cardiac surgery is limited. Palomba et al. suggested that the CVP value on ICU admission after cardiac surgery was independently related with AKI and a CVP greater than 14 cmH_2_O (about 10.3 mmHg) led to an approximately two-fold risk of AKI (27). In a retrospective study, Yang et al. reported that patients with CVP ≥ 10 mmHg after on-pump cardiac surgery had a 6-fold higher AKI incidence than those with CVP < 10 mmHg. They also found that CVP was an independent predictor of postoperative AKI and mortality among patients undergoing on-pump cardiac surgery (28).

However, the baseline characteristics of patients undergoing CABG and those undergoing valvular or aortic surgeries are significantly different. The prognostic value of postoperative CVP may deserve further investigation in CABG patients. A prospective study has investigated the association of prognosis and the clinical data collected in the early postoperative period after CABG. It was suggested that a CVP above 17 mmHg on ICU admission significantly increase the risk of postoperative complications and in-hospital mortality (29). Based on an analysis of 2001 CABG patients, Williams et al. suggested that CVP measured at 6 h after surgery was strongly associated with operative mortality and renal failure in CABG patients (30).

In the present study, we conducted a retrospective study among 3,564 patients underwent isolated CABG from the MIMIC-III database. In agreement with previous studies, the AKI incidence tended to be higher in patients with mean CVP above 10.9 mmHg in the first 24 h after CSRU admission. A curvilinear relationship of CVP with AKI incidence was observed in the present study. The incidence of AKI increased obviously since CVP above approximately 10 mmHg. The multivariate regression models indicated that CVP, especially above 10.9 mmHg, was an independent predictor of postoperative AKI. Guo et al. considered that the baseline characteristics between patients with or without elevated CVP were significantly different in previous studies, which may affect the reliability of the results (31). Such phenomenon can also be observed in the present study. Therefore, a PSM analysis was performed to alleviate the confounding effect of potential factors in our study. It was indicated that after PSM patients with CVP above 10.9 mmHg had significantly higher risk of postoperative AKI. In line with previous studies, higher in-hospital and 4-year mortality were observed in patients with higher CVP in the overall dataset. Also, CVP showed an independent association with in-hospital and 4-year mortality when it was selected as a continuous variable rather than a dichotomic variable, which might be attributed to the curvilinear relationship between CVP and mortality as shown in Figure 5. Unlike the AKI incidence, the incidence of in-hospital and 4-year mortality increased more obviously since CVP reached about 16 mmHg. Therefore, the transformation of CVP to a dichotomic variable based on the optimal cutoff value for AKI might be responsible for the results of multivariate regression analyses. In addition, in the PSM dataset, the differences of in-hospital and 4-year mortality between patients in the High CVP group and the Low CVP group were not statistically significant, and CVP, as a continuous or a dichotomic variable, was not independently associated with both in-hospital and 4-year mortality. We believed that these contradictory results might be assorted to the PSM for the optimal cutoff value of CVP for AKI determined by the ROC analysis. In summary, the mean value of CVP within the first 24 h after surgery was predictive of AKI and mortality in patients undergoing CABG. Especially, a CVP above 10.9 mmHg indicated about 50% higher risk of AKI. But as a dichotomic variable, CVP ≥ 10.9 mmHg was not significantly associated with both in-hospital and 4-year mortality. The optimal cutoff value of CVP for mortality and their association entail further investigation.

To our knowledge, this is the biggest cohort study to demonstrate the correlation between the postoperative CVP and the outcomes of CABG patients and the first study where PSM analysis was performed to balance the potential confounding factors. Besides, CVP is related to perioperative management and can vary dramatically in the early postoperative period. A 24-h average level of CVP may be more stable and able to diminish the confounding effect of postoperative management, compared to the value at an explicit timepoint. The present study indicated the association of CVP with postoperative AKI in CABG patients. Although CVP is influenced by multiple factors, like venous volume, cardiac function, intrathoracic pressure, and mechanical ventilation, we should revalue the CVP measurement for CABG patients in ICU during the early postoperative period, considering the pressure effect of CVP on kidneys (13). An elevated CVP might alarm clinicians to consider the etiology and adjust the management of vasoactive drugs and fluid administration to avoid the incidence of AKI after CABG (30). A personalized low CVP that assures adequate organ perfusion may be beneficial to CABG patients (13).

There are limitations in the present study. Firstly, the main limitation of the present study is its retrospective nature. The results of this study may be influenced by unmeasured variables. EuroScore, STS score and factors closely related to coronary artery disease (brain natriuretic peptide, cardiac troponin, and creatine kinase) and cardiac function (left ventricular ejection fraction) were not available in the present study. Secondly, creatinine level, urine output, and renal replacement therapy are included in the definition and staging of AKI according to the KDIGO guideline (9). We determined the incidence of AKI only according to the creatinine level, which may lead to an underestimation of AKI in the present study.

## Conclusion

In the present retrospective study, the mean value of CVP within the first 24 h after surgery was independently associated with AKI in patients undergoing CABG. Especially, a patient with mean CVP above 10.9 mmHg may warrant the clinicians to be aware of the risk of AKI.

## Data availability statement

The raw data supporting the conclusions of this article will be made available by the authors, without undue reservation.

## Ethics statement

The studies involving human participants were reviewed and approved by Massachusetts Institute of Technology (Cambridge, MA) and the Institutional Review Boards of Beth Israel Deaconess Medical Center (Boston, MA). Written informed consent for participation was not required for this study in accordance with the national legislation and the institutional requirements.

## Author contributions

SZ, XW, and ZX contributed to the conception and design of the study, and revised the manuscript. JL and RW were responsible for the data extraction, performed the statistical analysis, wrote the manuscript, and created the figures and tables. PZ and JW participated in drafting the manuscript. All authors read and approved the final manuscript.

## Abbreviations

AKI: Acute kidney injury
AUC: Area under the curve
BMI: Body mass index
BUN: Blood urea nitrogen
CABG: Coronary artery bypass grafting
CCI: Charlson comorbidity index
CI: Confidence interval
CPB: Cardiopulmonary bypass
CSRU: Cardiac surgery recovery unit
CVP: Central venous pressure
DBP: Diastolic blood pressure
HR: Hazard ratio
ICD9-CM: International Classification of Diseases Ninth Revision Clinical Modification
ICU: Intensive care unit
IMA: Internal mammary artery
KDIGO: Kidney Disease Improving Global Outcomes
MBP: Mean blood pressure
MIMIC-III: Medical Information Mart for Intensive Care III
OR: Odds ratio
ROC: Receiver operating characteristic
PSM: Propensity score matching
SBP: Systolic blood pressure
SOFA: Sequential organ failure assessment.
