# Supplementary material for: Postoperative central venous pressure is associated with acute kidney injury in patients undergoing coronary artery bypass grafting
Source: Front Cardiovasc Med. 2022 Nov 17;9:1016436. doi: 10.3389/fcvm.2022.1016436 (PMC9712208; doi:10.3389/fcvm.2022.1016436)
Supplement: Supplementary file 1 [file Data_Sheet_1.docx]

Supplementary Table 1. Association of postoperative CVP with AKI and mortality in patients undergoing on-pump CABG.

| Outcome | Variable | OR/HR (95%CI) | | | | |
| --- | --- | --- | --- | --- | --- | --- |
|  |  | Unadjusted | Model 1 | Model 2 | Model 3 | Model 4 |
| AKI | CVP (mmHg) | 1.122 (1.092, 1.153) | 1.120 (1.088, 1.153) | 1.103 (1.069, 1.137) | 1.101 (1.068, 1.136) | 1.064 (1.026, 1.102) |
|  | CVP ≥ 10.9 mmHg | 1.876 (1.587, 2.219) | 1.892 (1.583, 2.261) | 1.750 (1.449, 2.113) | 1.739 (1.439, 2.100) | 1.459 (1.188, 1.791) |
| In-hospital mortality | CVP (mmHg) | 1.478 (1.348, 1.621) | 1.466 (1.332, 1.612) | 1.384 (1.234, 1.553) | 1.360 (1.214, 1.524) | 1.186 (1.017, 1.382) |
|  | CVP ≥ 10.9 mmHg | 5.917 (2.421, 14.462) | 5.765 (2.305, 14.421) | 3.875 (1.455, 10.316) | 3.788 (1.409, 10.184) | 1.227 (0.366, 4.108) |
| 4-year mortality | CVP (mmHg) | 1.118 (1.077, 1.161) | 1.145 (1.102, 1.190) | 1.119 (1.076, 1.164) | 1.115 (1.073, 1.160) | 1.074 (1.023, 1.128) |
|  | CVP ≥ 10.9 mmHg | 1.554 (1.225, 1.972) | 1.798 (1.403, 2.304) | 1.671 (1.292, 2.163) | 1.668 (1.289, 2.160) | 1.271 (0.947, 1.706) |

Model 1 adjusted for age, gender, BMI.

Model 2 adjusted for variables in Model 1, plus comorbidities (cardiac arrhythmias, congestive heart failure, peripheral vascular disease, myocardial infarction, chronic pulmonary disease, liver disease, renal disease, diabetes mellitus) and laboratory examinations (BUN, creatinine, white blood cell, platelet, hemoglobin) on admission.

Model 3 adjusted for variables in Model 2, plus surgery-related variables.

Model 4 adjusted for variables in Model 3, plus variables related to the clinical situation in CSRU, including vitals, SOFA score, net fluid input and erythrocyte transfusion within the first 24 hours after CSRU admission.

AKI: Acute kidney injury. BMI: Body mass index. BUN: Blood urea nitrogen. CABG: Coronary artery bypass grafting. CI: Confidence interval. CSRU: Cardiac surgery recovery unit. CVP: Central venous pressure. HR: Hazard ratio. OR: Odds ratio. SOFA: Sequential organ failure assessment.

Supplementary Table 2. Association of postoperative CVP with AKI and mortality in patients undergoing off-pump CABG.

| Outcome | Variable | OR/HR (95%CI) | | | | |
| --- | --- | --- | --- | --- | --- | --- |
|  |  | Unadjusted | Model 1 | Model 2 | Model 3 | Model 4 |
| AKI | CVP (mmHg) | 1.247 (1.131, 1.373) | 1.220 (1.096, 1.358) | 1.246 (1.105, 1.404) | 1.240 (1.098, 1.400) | 1.202 (1.047, 1.381) |
|  | CVP ≥ 10.9 mmHg | 2.900 (1.600, 5.256) | 2.639 (1.384, 5.031) | 2.889 (1.427, 5.851) | 2.804 (1.376, 5.714) | 2.641 (1.201, 5.808) |
| In-hospital mortality^*^ | CVP (mmHg) | - | - | - | - | - |
|  | CVP ≥ 10.9 mmHg | - | - | - | - | - |
| 4-year mortality | CVP (mmHg) | 1.051 (0.947, 1.167) | 1.016 (0.912, 1.131) | 0.984 (0.867, 1.116) | 1.011 (0.886, 1.153) | 0.986 (0.841, 1.155) |
|  | CVP ≥ 10.9 mmHg | 0.984 (0.523, 1.853) | 0.817 (0.422, 1.583) | 0.776 (0.356, 1.693) | 0.880 (0.399, 1.941) | 0.963 (0.380, 2.438) |

Model 1 adjusted for age, gender, BMI.

Model 2 adjusted for variables in Model 1, plus comorbidities (cardiac arrhythmias, congestive heart failure, peripheral vascular disease, myocardial infarction, chronic pulmonary disease, liver disease, renal disease, diabetes mellitus) and laboratory examinations (BUN, creatinine, white blood cell, platelet, hemoglobin) on admission.

Model 3 adjusted for variables in Model 2, plus surgery-related variables.

Model 4 adjusted for variables in Model 3, plus variables related to the clinical situation in CSRU, including vitals, SOFA score, net fluid input and erythrocyte transfusion within the first 24 hours after CSRU admission.

AKI: Acute kidney injury. BMI: Body mass index. BUN: Blood urea nitrogen. CABG: Coronary artery bypass grafting. CI: Confidence interval. CSRU: Cardiac surgery recovery unit. CVP: Central venous pressure. HR: Hazard ratio. OR: Odds ratio. SOFA: Sequential organ failure assessment.

^*^ None of patients died in hospital.
